# Atox1 regulates macrophage polarization in intestinal inflammation via ROS-NLRP3 inflammasome pathway

**DOI:** 10.1186/s12967-024-05314-4

**Published:** 2024-05-25

**Authors:** MingXian Chen, Yu Chen, Rui Fu, SaiYue Liu, HaiXia Li, TangBiao Shen

**Affiliations:** 1 Department of Gastroenterology, Tongde Hospital of Zhejiang Province, No. 234, Gucui road, Hangzhou, 310012, China; 2 Institute of Integrated Chinese and Western Medicine on Spleen-Stomach Diseases, Zhejiang Province Academy of Traditional Chinese Medicine, Hangzhou, 310012, China; 3 Laboratory Animal Center, Zhejiang Province Academy of Traditional Chinese Medicine, Hangzhou, 310012, China; 4 Department of Adverse Drug Reaction Monitoring, Zhejiang Province Center of Adverse Drug Reaction Monitoring, No. 39, Yile road, Hangzhou, 310012, China; 5 Department of Cardiology, Guanganmen Hospital of China Academy of Chinese Medical Sciences, No. 5, Beixian Ge, Xicheng District, Beijing, 100053, China

**Keywords:** Inflammatory bowel disease, Copper metabolism, Macrophage polarization, ROS, NLRP3 inflammasome

## Abstract

**Background:**

Inflammation and oxidative stress play an important role in the pathophysiology of inflammatory bowel disease (IBD). This study aimed to explore the effects of copper chaperone Antioxidant-1 (Atox1) on macrophages in a mouse model of intestinal inflammation.

**Methods:**

A mouse model of TNBS-induced colitis was established and verified using the disease activity index. Atox1 conditional knockout mice were applied. The proportion of macrophages in colonic lamina propria mononuclear cells and ROS production were analyzed using flow cytometry. Inflammatory cytokines were measured using ELISA. Expression of macrophage M1/M2 polarization markers, p47phox, NLRP3, and Caspase-1 p20 was measured using quantitative RT-PCR and Western blotting.

**Results:**

Atox1 expression was up-regulated in colon tissues of TNBS-induced colitis mice. Macrophages isolated from TNBS-induced colitis mice showed M1 polarization and nuclear translocation of Atox1. Inhibiting copper chaperone activity decreased p47phox, ROS production, and M1 polarization induced by CuCl_2_ in macrophages. TNBS induced up-regulation of inflammatory cytokines, M1 polarization markers, and p47phox expression in mice, an effect which was preempted by Atox1 knockout. Inflammatory cytokines and expression of M1 polarization markers, p47phox, NLRP3, Caspase-1 p20 were also increased in macrophages isolated from TNBS-induced colitis mice. These changes were alleviated in mice with Atox1 knockout. The effects of Atox1 on macrophage polarization were mediated via the ROS-NLRP3 inflammasome pathway.

**Conclusion:**

Atox1 plays a pro-inflammatory role, promotes M1 polarization of macrophages, and increases the concentrations of pro-inflammatory cytokines in intestinal tissue by regulating the ROS-NLRP3 inflammasome pathway. Atox1 is a potential therapeutic target in IBD.

**Supplementary Information:**

The online version contains supplementary material available at 10.1186/s12967-024-05314-4.

## Introduction

Inflammatory bowel disease (IBD), including ulcerative colitis and Crohn’s disease, is a chronic, potentially life-threatening inflammatory disorder of the digestive tract [1, 2]. IBD has a complex pathophysiology involving the interplay of immunological, environmental, microbial, and genetic factors [3, 4]. There is a growing appreciation that oxidative stress and inflammatory responses contribute to the occurrence and progression of IBD, affecting mitochondrial function [5, 6]. Thus, the use of antioxidants to scavenge reactive oxygen species (ROS) and suppress inflammation is considered a therapeutic strategy for IBD [7].

Inflammation boosts the uptake of copper (Cu), leading to increased Cu levels in inflamed colon tissues [8]. Cu induces inflammation and microglia activation through the ROS/NF-κB pathway [9]. Antioxidant-1 (Atox1) is a small metallochaperone protein that plays a crucial role in intracellular Cu trafficking and homeostasis [10]. Atox1 provides copper to P-type ATPases that transport copper, which are essential for the proper functioning of numerous cellular processes, including redox homeostasis, energy production, and cell signaling [11]. Atox1 is also a crucial regulator of cell proliferation, cell cycle, metastasis, and DNA repair and represents a potential target in cancer therapy including colon cancer [12–14]. Moreover, in a study, Atox1 expression was found to be higher in active ulcerative colitis samples, was negatively correlated with CD8^+^ T cell infiltration, and showed excellent diagnostic value for ulcerative colitis [15]. Atox1 up-regulates ROS production in endothelial cells by regulating the NOX organizer p47phox, and thus, may be a therapeutic target in inflammatory diseases such as atherosclerosis [16]. However, the effects of Atox1-mediated inflammation in the progression of Crohn’s disease and the underlying mechanisms are not well characterized.

In addition to inflammation, an imbalance of M1/M2 macrophage polarization is another important factor in the pathophysiology of IBD [17]. M1 subtype macrophages produce pro-inflammatory cytokines, such as IL-6, TNF-α, and IL-1β, while M2 subtype macrophages suppress inflammation [18]. M1 and M2 subtypes macrophages are increasingly acknowledged as potential therapeutic targets for IBD [19]. Moreover, Cu has been shown to promote M1 polarization of macrophages by increasing ROS production [20], and this effect may vary depending on the concentration of Cu [21]. However, the effects of copper chaperone Atox1 on macrophage polarization and the underlying signaling pathways remain largely uncharacterized.

To fill the knowledge gap, in the current study, we detected Atox1 expression and M1/M2 macrophage polarization in colon tissues of trinitrobenzene sulfonic acid (TNBS)-induced mice models. The TNBS-induced experimental mouse model exhibits a colitis-like phenotype with histopathological and morphological changes similar to that in Crohn’s disease [22, 23]. Using copper chaperone inhibitor and Atox1-knockout mice, we explored the impact of Atox1 on ROS production, M1/M2 macrophage polarization, and inflammation. Our findings suggest a “copper-Atox1-ROS-NLRP3 inflammasome” regulatory axis in inflammatory disorders and demonstrate that Atox1 may regulate macrophage polarization, suggesting its potential as a therapeutic target for IBD and highlighting the importance of Atox1 as a key mediator in the complex network of inflammatory processes.

## Materials and methods

### Ethical approval

The study was approved by the Ethics Committee of the Zhejiang Academy of Traditional Chinese Medicine ([2023]043). All animal experiments were conducted in accordance with the Guide for the Care and Use of Laboratory Animals.

### Bioinformatics analysis

The single-cell RNA-seq data of patients with Crohn’s disease were downloaded from the GEO database (accession number: GSE134809). The unique molecular identifier (UMI) count matrix was converted to anndata objects using Scanpy package v1.4.4. Batch correction was performed using the RunHarmony function in its R package (version 1.0). Uniform Manifold Approximation and Projection (UMAP) was used for data visualization. The effect size was evaluated using Cohen’s d statistic to estimate the magnitude of differentially expressed genes.

### Experimental animals

The Atox1 knockout mouse model (C57BL/6J) was generated by CRISPR/Cas-mediated genome engineering (Cyagen Biosciences) through the insertion of the LoxP site flanking exon 2 of the *Atox1* gene. The Atox1 heterozygous flox-labeled mice (Atox1^+/−^) were backcrossed with C57BL/6J mice five times to obtain F1 generation positive mice stably expressing the allele. A different litter of Atox1^+/−^ mice was crossed to obtain the homozygous floxed Atox1 allele (Atox1^−/−^). Next, the sexually mature *Atox1* knockout mice were mated with Lyz2^Cre^ mice (Cyagen Biosciences) to establish mice with mutated *Atox1*, specifically in the macrophage.

### Animal experimental design and establishment of TNBS-induced colitis model

Male C57BL/6J mice (age: 7–8 weeks, SPF grade) were purchased from Cyagen Biosciences. Mice were randomly assigned to following groups (6 mice/group), i.e., wild type (WT) mice as control, WT mice treated with 2,4,6-Trinitrobenzenesulfonic acid (TNBS), Atox1^−/−^ mice, Atox1^−/−^ mice treated with TNBS (Atox1^−/−^+TNBS), Atox1^+/−^ mice, Atox1^+/−^ mice treated with TNBS (Atox1^+/−^+TNBS), and Atox1^+/−^+TNBS mice treated with Cu chaperone inhibitor DCAC50 (Atox1^+/−^+TNBS + DCAC50). The detailed procedure for induction of colitis is described elsewhere [24]. Briefly, after overnight fasting, a single dose of TNBS (4 mg in 0.25 mL of 50% ethanol) was administered to the mice intracolonically under anesthesia for 96 h. To evaluate the therapeutic effect of DCAC50 on TNBS-induced colitis, Atox1 conditional knockout mice received intragastrical DCAC50 for 72 h (50 mg/kg/d; Sigma-Aldrich) [25] before TNBS and 72 h after TNBS induction. Mice were examined daily for diarrhea and rectal bleeding. Disease activity index (DAI) was determined by scoring changes in body weight, presence of blood in stool, and stool consistency, as described previously [26]. After 72–96 h of TNBS instillation, the mice were euthanized, and the colon tissues were collected.

### Hematoxylin and eosin (H&E) and immunofluorescence staining

The colon tissues were fixed in a 4% paraformaldehyde solution for 24 h and then dehydrated and embedded in paraffin. Subsequently, 4-µm thick sections were prepared. The sections were deparaffinized in xylene, and hydrated by passage through graded alcohol series (decreasing concentrations). The sections were stained with H&E. For immunofluorescence staining assay, the sections were incubated in 3% H_2_O_2_ (Sigma–Aldrich) at 37 °C for 30 min, and washed with phosphate-buffered saline (PBS; Gibco). Subsequently, sections were boiled in 0.01 M citric acid buffer at 95 °C for 20 min, cooled to room temperature, and washed with PBS. Blocking was performed with 5% bovine serum albumin for 1 h at room temperature. Sections were stained overnight with anti-Atox1 (1:100; Invitrogen, MA5-18452) or anti-F4/80 (1:50; Abcam, ab300421) antibody at 4 °C, followed by Alexa Fluor 555-labeled Donkey Anti-Rabbit IgG (H + L) (1:200; Beyotime Biotechnology, A0453) or Alexa Fluor 488-labeled Goat Anti-Mouse IgG (H + L) (1:200; Beyotime Biotechnology, A0428) antibody for 1 h at room temperature. Next, diluted DAPI (4′,6-diamidino‐2‐phenylindole) was added and the sections were incubated in the dark for approximately 15 min. Positively stained cells were visualized using a confocal laser scanning microscope (Leica Microsystems).

### Flow cytometry analyses

After 72 h of TNBS instillation, colonic lamina propria mononuclear cells were isolated from colon tissues of TNBS-induced colitis mice as previously described [27] and stained with anti-CD45, anti-CD11b, anti-F4/80, and anti-Ly6C antibodies for flow cytometry analysis [28]. CD45^+^CD11b^+^F4/80^+^Ly6C^−^ cells were considered as macrophages. The cells were analyzed using a flow cytometer (Beckman Coulter, Brea, CA, USA).

### Isolation and treatment of murine macrophages

Macrophages were obtained from the intestinal mucosa of mice as previously described [27] and laid over 6-well culture plates (2 × 10^6^ cells/well) in RPMI 1640 media containing 10% FBS and 1% penicillin/streptomycin at 37 °C with 5% CO_2_. Macrophages were pre-treated with 20 µM DCAC50 for 24 h, followed by treatment with 100 µM copper (II) chloride dihydrate (CuCl_2_; Wako Pure Chemicals, Osaka, Japan) for 4 h. Otherwise, macrophages were treated with 1 mM of ROS scavenger N-acetyl cysteine (NAC; Selleck) for 24 h or 5 µM of NLRP3 specific inhibitor MCC950 (Selleck) for 24 h.

### Cu measurements

Copper levels in colon tissue were determined using a copper colorimetric assay kit (E-BC-K300-M; Elabscience Biotechnology Co. Ltd.). 20 µL of colon tissue homogenate was added to a 96-well plate, and 300 µL of detection reagent was mixed. The intracellular copper content was measured by Cell Copper (Cu) Colorimetric Assay Kit (E-BC-K775-M; Elabscience Biotechnology Co. Ltd.). 2 × 10^6^ macrophages were mixed with 200 µL of lysis buffer for 10 min on ice. Subsequently, macrophages were centrifugated at 12,000 g for 10 min at 4 °C, and the cell supernatant was collected. Then, 100 µL of cell supernatant was added into wells of a 96-well plate, and 50 µL of detection reagent was mixed. The plate was incubated at 37 °C for 5 min and optical density was read at 580 nm using a microplate reader.

### Measurement of ROS

ROS levels within the macrophages were assessed using a fluorescent DCFH-DA probe (Beyotime Biotechnology). Briefly, the DCFH-DA probe was added into the cell culture medium to a final concentration of 10 µM. After incubation for 20 min in the dark at 4 °C, the fluorescence was determined using flow cytometry.

### Quantitative RT-PCR

Total RNA was isolated from the tissue homogenate and macrophages using TRIzol® reagent (Thermo Fisher Scientific, Inc.). cDNA was synthesized using the PrimeScript kit (Takara Biotechnology). The cDNA synthesis conditions were 37 °C for 60 min, followed by 85 °C for 5 min and 4 °C for 5 min. Quantitative RT-PCR using SYBR green PCR master mix (Applied Biosystems) was performed using an ABI 7500 real-time PCR system (Applied Biosystem). The PCR cycling conditions were as follows: 95 °C for 10 min followed by 40 cycles at 95 °C for 15 s and 60 °C for 45 s followed by a final extension step of 95 °C for 15 s, 60 °C for 1 min, 95 °C for 15 s, and 60 °C for 15 s. The primers used for PCR are listed in Table 1. The relative expressions of Nos2, Il12b, Il10, Arg1, and p47phox were obtained using the 2^−ΔΔCt^ method by designating Actb as the control gene.

**Table 1:** The primer sequences used in the study

Gene	Forward/Reverse	Sequences
Mus musculus Nos2	Forward	5ʹ-GAGCAACTACTGCTGGTGGT-3ʹ
	Reverse	5ʹ-CGATGTCATGAGCAAAGGCG-3ʹ
Mus musculus Il12b	Forward	5ʹ-AGTGACATGTGGAATGGCGT-3ʹ
	Reverse	5ʹ-CAGGAGTCAGGGTACTCCCA-3ʹ
Mus musculus Il10	Forward	5ʹ-GTAGAAGTGATGCCCCAGGC-3ʹ
	Reverse	5ʹ-TAGACACCTTGGTCTTGGAGC-3ʹ
Mus musculus Arg1	Forward	5ʹ-TCGGAGCGCCTTTCTCAAAA-3ʹ
	Reverse	5ʹ-CACAGACCGTGGGTTCTTCA-3ʹ
Mus musculus p47phox	Forward	5ʹ-CGTTCTCGGAAGCGCCTTAG-3ʹ
	Reverse	5ʹ-GGATTGTCTCTGCCCTCCAGC-3ʹ
Mus musculus Actb	Forward	5ʹ-GCGTGACATCAAAGAGAAGC-3ʹ
	Reverse	5ʹ-ATGCCACAGGATTCCATACC-3ʹ

### Western blot analysis

Protein was extracted from the tissue homogenate and macrophages using RIPA lysis buffer (Sigma-Aldrich). NE-PER™ Nuclear and Cytoplasmic Extraction Reagents (Thermo Fisher Scientific) were used to prepare the cytosolic fraction and nuclear extracts. Equivalent quantities (25 µg) of protein were separated by SDS-PAGE gel, transferred onto a nitrocellulose membrane (Millipore), blocked with 5% skim milk overnight at 4 °C, followed by overnight incubation with primary antibodies against Atox1 (1:10000; Abcam, ab154179), p47phox (1:1000; Abcam, ab181090), NLRP3 (1:500; Abcam, ab263889), Caspase 1 p20 (1:1000; Invitrogen, PA5-99390), iNOS (1:20000; Abcam, ab178945), IL-12p40 (1:1000; Abcam, ab133752), IL-10 (1:2000; Abcam, ab1333575), Arg-1 (1:1000; Abcam, ab2333548), Lamin B1 (1:1000; Abcam, ab229025), and β-actin (1:5000; Proteintech Group, Inc., 60066-1-AP) at 4 °C. Subsequently, the membranes were washed thrice with Tris-buffered saline with 0.1% Tween-20 and incubated with the HRP-conjugated secondary antibody (1:10000; ZSGB-BIO, ZB-2301, ZB-2305) for 1 h at 37 °C. Signals were visualized with an enhanced chemiluminescence system.

### ELISA

The colon tissues were homogenized in PBS on ice and then centrifugated at 500 g for 10 min at 4 °C and the supernatant was collected and analyzed. The cell culture medium was centrifugated at 1000 g for 20 min at 4 °C to remove impurities and cell debris, and the supernatant was collected and analyzed. The levels of TNF-α (E-EL-M3063; Elabscience, Houston, TX, USA), IFN-γ (E-EL-M0048c; Elabscience), IL-6 (E-EL-M0044c; Elabscience), IL-1β (E-EL-M0037c; Elabscience), IL-18 (E-EL-M0730c; Elabscience), and IL-17 (BMS6001TEN; Invitrogen) were determined using commercial ELISA kits. The optical density was measured at 450 nm using a microplate reader.

### Luciferase reporter assays

Mus musculus p47phox (NM_001286037.1) promoter fragment (676 bp) spanning from − 593 to + 82 nt of the p47phox transcription start site was amplified by primers (Forward 5ʹ-GAAGCTGGCCTTGAACCCTT-3ʹ, Reverse 5ʹ-ATGGCGAATGAAGGTGTCCC-3ʹ) and cloned into pGL3-basic vectors (Promega). The pGL3-p47phox promoter reporter vector was transfected into cells using Lipofectamine 3000 (Invitrogen). After 48 h, the luciferase activity was evaluated using the Dual-Luciferase Reporter Assay System (Promega) as per the manufacturer’s recommendations.

### Chromatin immunoprecipitation (ChIP) assays

ChIP assays were conducted using the Magnetic ChIP kit (Millipore), according to the manufacturer’s instructions. Cells were immobilized using 1% formaldehyde and fragmented in the presence of MNase and by sonication. Rabbit polyclonal antisera to Atox1 [29] and rabbit IgG control polyclonal antibody (Proteintech, 30000-0-AP) were used for immunoprecipitation assays. After washing and reverse-crosslinking, the precipitated DNA was amplified by primers (Forward 5ʹ-GCCATGGTGTCGGTAGAACA-3ʹ, Reverse 5ʹ-CCGTGACAGGGACACTTCTC-3ʹ) and quantified by PCR.

### Statistical analysis

All experiments were performed in triplicate and quantitative data were expressed as mean ± standard deviation (SD). Between-group differences were assessed for statistical significance using the Student’s t-test, while ANOVA followed by Dunnett’s multiple comparison test was used for multi-group comparisons. All statistical analyses were performed using GraphPad Prism 8.4.2 (GraphPad Software). *P* values < 0.05 were considered indicative of statistical significance.

## Results

### Atox1 expression was up-regulated in TNBS-induced colitis mice

**Fig. 1:**
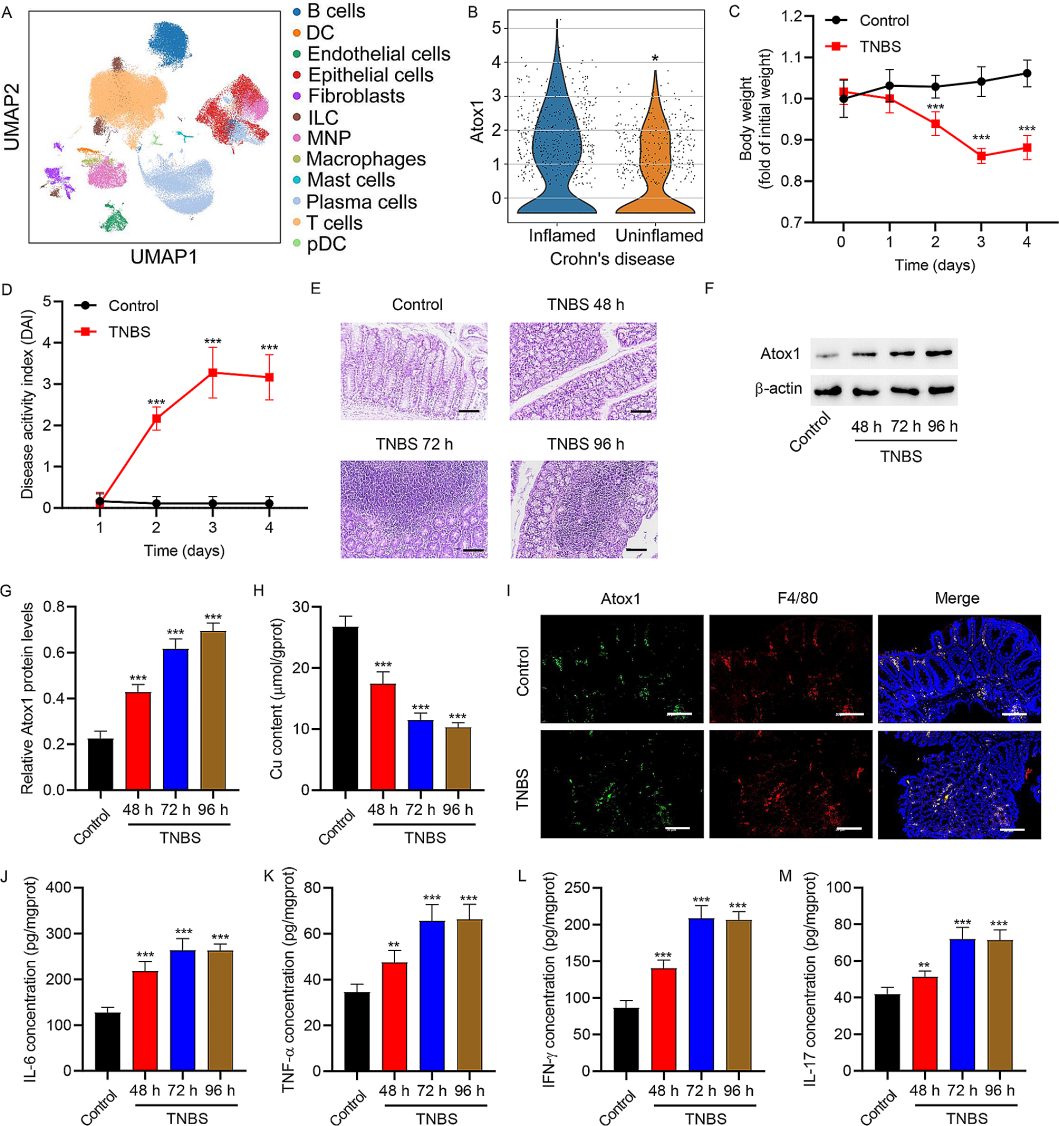
Atox1 expression was increased in TNBS-induced colitis mice. (**A**) Uniform Manifold Approximation and Projection (UMAP) plot of cell types in patients with Crohn’s disease (GSE134809). (**B**) Violin plot showing the expression of Atox1 in macrophages from the inflamed and uninflamed areas of patients with Crohn’s disease. TNBS-induced colitis mice were evaluated by (**C**) body weight loss, expressed as a percentage of the initial weight, and (**D**) clinical disease activity index (DAI). (**E**) Representative images of histological analysis in TNBS-induced colitis mice. (**F**, **G**) Western blot analyses of Atox1 expression in colonic tissues of TNBS-induced colitis mice. (**H**) Biochemical analysis of Cu content in colonic tissues of TNBS-induced colitis mice. (**I**) Representative images of immunofluorescence analyses of colonic mucosa in control and TNBS-induced colitis mice showing F4/80 + cells and Atox1 + cells. The merged images show co-localization of Atox1 with F4/80. Results of ELISA showing the mucosal production of the pro-inflammatory cytokines (**J**) IL-6, (**K**) TNF-α, (**L**) IFN-γ, and (**M**) IL-17 in control and TNBS-induced colitis mice. DC, dendritic cell; ILC, innate lymphoid cell; MNP, mononuclear phagocyte; pDC, plasmacytoid dendritic cell. Scale bar, 100 μm. *P < 0.05, ***P < 0.001 vs. inflamed or control

The single-cell RNA-seq data of patients with Crohn’s disease were downloaded from the GEO database. The UMAP plot of cell types in patients with Crohn’s disease is shown in Fig. 1A, in which macrophages were identified by expressing MERTK, CTSC, CTSD, GLUL, PLD3, CD14, CD68, and FCGR3A [30]. Atox1 expression in macrophages was elevated in the inflamed areas compared to the uninflamed area of the colon in patients with Crohn’s disease (Fig. 1B). Next, colitis mice models were induced by TNBS for 4 days. As shown in Fig. 1C, model mice showed a significant decrease in body weight than control mice on days 2, 3, and 4. The disease activity index (DAI) was also significantly increased in the model mice compared to the control mice on days 2, 3, and 4 (Fig. 1D). Histological examination of the colonic tissue of model mice showed significantly decreased villus height, disrupted crypt architecture, and obvious signs of inflammation (Fig. 1E). Moreover, the colonic tissue of model mice showed up-regulation of Atox1 protein expression and a decrease in Cu content (Fig. 1F-H). Immunofluorescent images showed the co-localization of Atox1 with macrophages at day 3 after TNBS induction (Fig. 1I). Moreover, the levels of pro-inflammatory cytokines (IL-6, TNF-α, IFN-γ, and IL-17) in colonic tissues were significantly elevated on day 3 after TNBS induction (Fig. 1J-M).

### TNBS induction regulated M1/M2 macrophage polarization and up-regulated Atox1 nuclear translocation

**Fig. 2:**
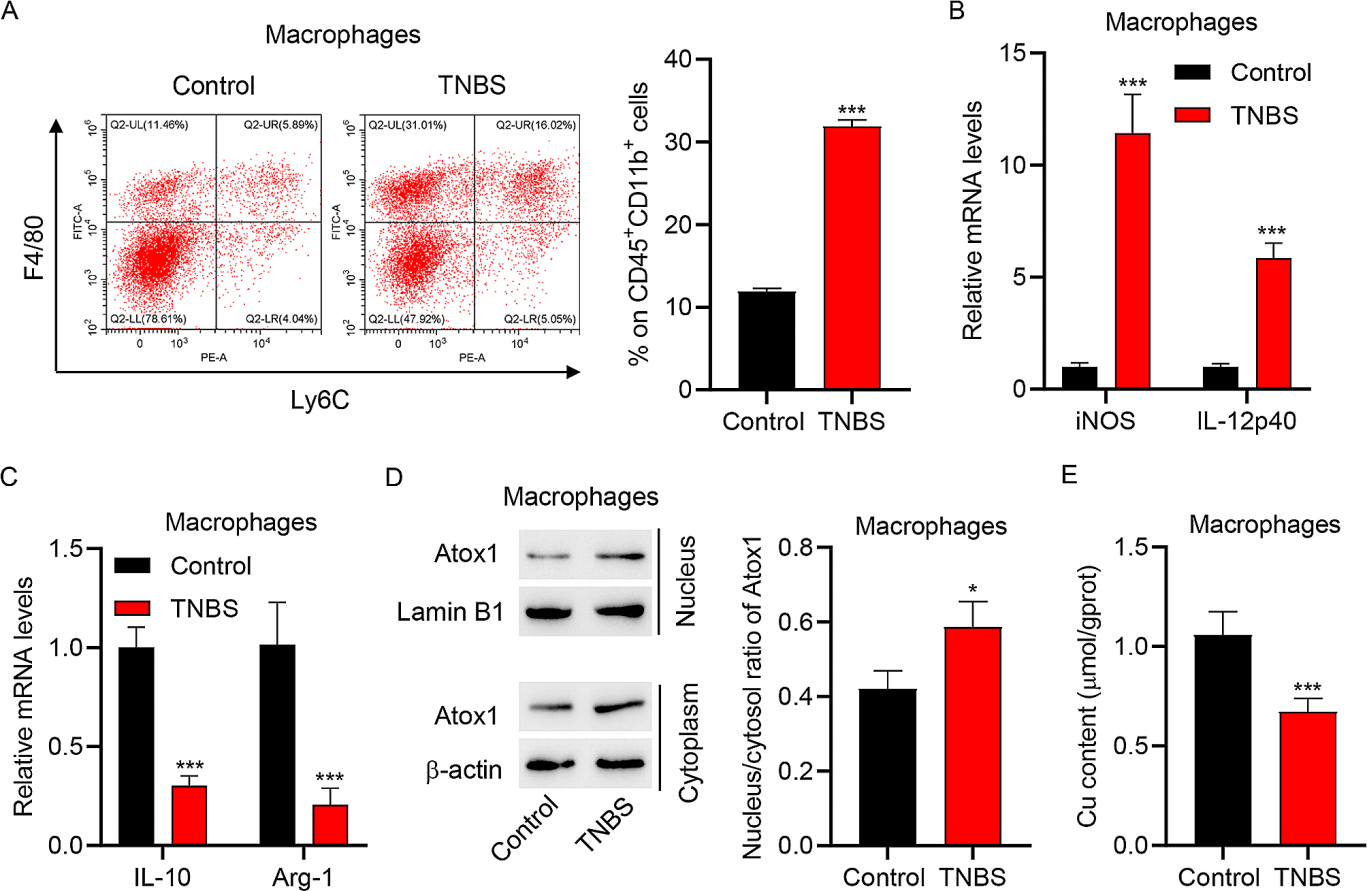
Macrophage polarization in TNBS-induced colitis mice. (**A**) Results of flow cytometry showing the percentages of macrophages (CD45^+^CD11b^+^F4/80^+^Ly6C^−^) among the colonic lamina propria mononuclear cells isolated from control and TNBS-induced colitis mice. Results of quantitative RT-PCR showing the mRNA expressions of (**B**) M1 polarization markers (iNOS and IL-12p40) and (**C**) M2 polarization markers (IL-10 and Arg-1) in the macrophages isolated from the intestinal mucosa of control and TNBS-induced colitis mice. (**D**) Western blot assay to determine Atox1 expression and (**E**) biochemical analysis of Cu content in macrophages isolated from the intestinal mucosa of control and TNBS-induced colitis mice. *P < 0.05, ***P < 0.001 vs. control

We investigated the characteristics of macrophage polarization in TNBS-induced colitis mice. The percentage of macrophages in colonic lamina propria mononuclear cells isolated from TNBS-induced colitis mice was markedly increased compared with control mice (Fig. 2A). Macrophages isolated from the intestinal mucosa of TNBS-induced colitis mice showed increased mRNA levels of iNOS and IL-12p40 (M1 polarization markers) and decreased mRNA levels of IL-10 and Arg-1 (M2 polarization markers) compared with control mice (Fig. 2B-C). Moreover, macrophages isolated from the intestinal mucosa of TNBS-induced colitis mice showed upregulation of Atox1 nuclear translocation and a decrease in Cu content (Fig. 2D-E). These results indicated that TNBS induction increased macrophage percentage, promoted M1 polarization, inhibited M2 polarization, and up-regulated nuclear translocation of Atox1.

### Copper chaperone inhibitor DCAC50 suppressed CuCl_2_-induced ROS production and M1 polarization of macrophages

**Fig. 3:**
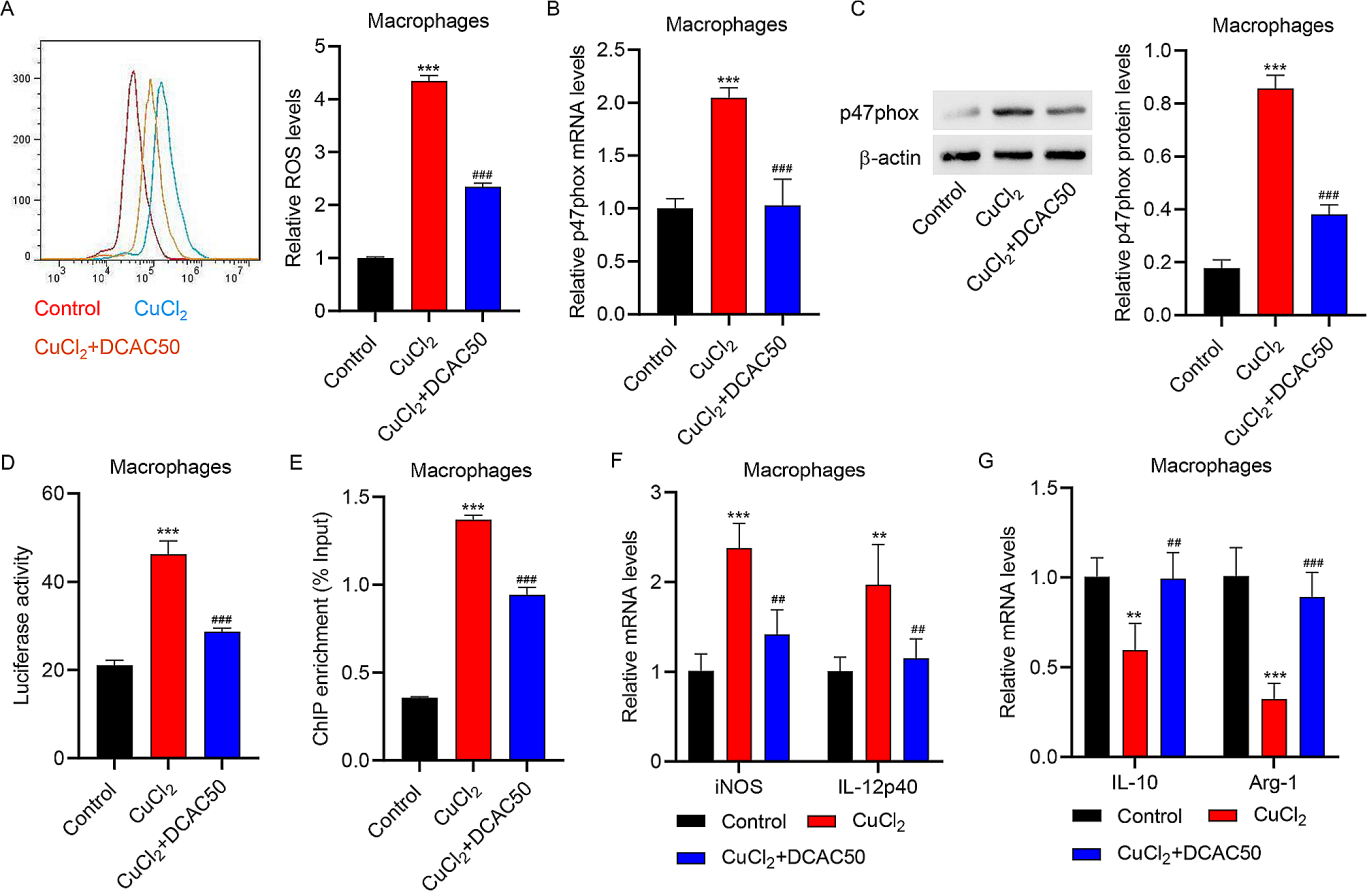
DCAC50 reduced CuCl_2_-induced ROS production and M1 polarization in macrophages. Macrophages isolated from the intestinal mucosa of mice were pre-treated with DCAC50, followed by CuCl_2_ treatment. (**A**) Results of flow cytometry to determine ROS production. (**B**) Results of quantitative RT-PCR and (**C**) Western blot assay to estimate mRNA and protein expression of p47phox. (**D**) Luciferase activity and (**E**) ChIP-PCR assays to measure the binding of p47phox promoter with Atox1. Results of quantitative RT-PCR to determine the mRNA expressions of (**F**) iNOS, (**F**) IL-12p40, (**G**) IL-10 and (**G**) Arg-1. **P < 0.01, ***P < 0.001 vs. control. ##*P* < 0.01, ###*P* < 0.001 vs. CuCl_2_

To explore the effect of Atox1 on ROS production in the macrophages isolated from the intestinal mucosa of mice, macrophages were treated with CuCl_2_ in the absence or presence of DCAC50, a recently developed small-molecule copper chaperone inhibitor [25]. As shown in Supplementary Fig. 1, CuCl_2_ treatment up-regulated Atox1 nuclear translocation in macrophages. CuCl_2_ treatment increased ROS accumulation, an effect that was largely inhibited by DCAC50 pretreatment (Fig. 3A). CuCl_2_ treatment up-regulated the mRNA and protein expressions of p47phox, whereas DCAC50 pretreatment largely alleviated these changes (Fig. 3B-C). Next, a luciferase reporter assay was conducted to explore the relationship between Atox1 and p47phox. The results showed that CuCl_2_ treatment increased the activity of the p47phox promoter, which was decreased by DCAC50 (Fig. 3D). ChIP-PCR assay showed that the binding between the p47phox promoter and Atox1 was increased by CuCl_2_ and decreased by DCAC50 (Fig. 3E).

Moreover, CuCl_2_ treatment led to increased mRNA levels of iNOS and IL-12p40 but decreased mRNA levels of IL-10 and Arg-1; these effects were partly inhibited by DCAC50 pretreatment (Fig. 3F-G). These results suggest that inhibiting copper chaperone activity counteracts the effects of CuCl_2_ on ROS production and macrophage polarization in colitis.

### Atox1 knockout suppressed the progression of TNBS-induced colitis by decreasing pro-inflammatory cytokines and inhibiting M1 polarization of macrophages

**Fig. 4:**
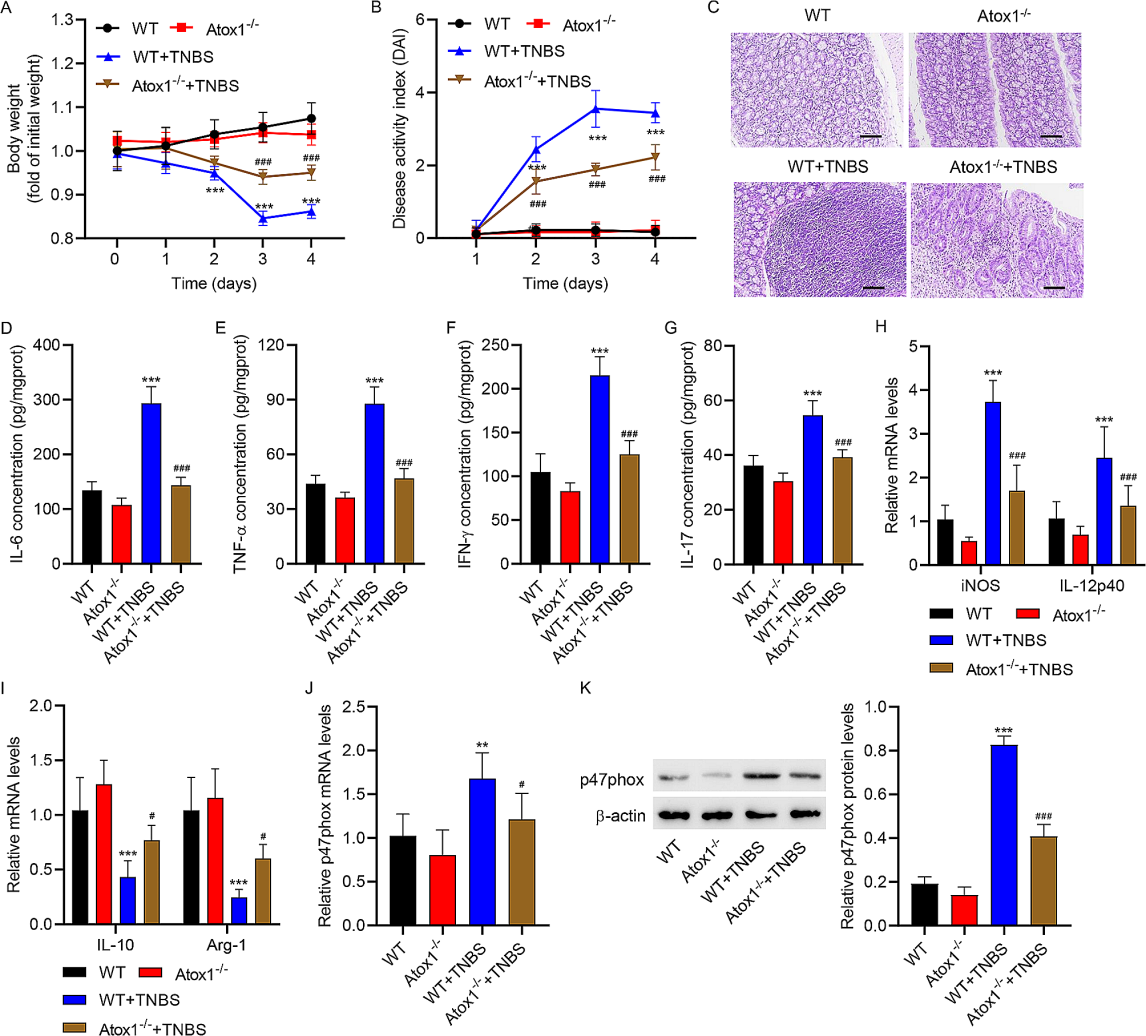
Atox1 knockout decreased the levels of pro-inflammatory cytokines after TNBS-induced colitis. Atox1^−/−^ mice and wild-type (WT) littermates received TNBS. Colitis induction was evaluated by (**A**) body weight loss, expressed as a percentage of the initial weight, and (**B**) clinical DAI. (**C**) Representative images of histological analysis. Results of ELISA showing the mucosal production of the pro-inflammatory cytokines (**D**) IL-6, (**E**) TNF-α, (**F**) IFN-γ, and (**G**) IL-17. Results of quantitative RT-PCR showing the mucosal mRNA expression of (**H**) iNOS, (**H**) IL-12p40, (**I**) IL-10 and (**I**) Arg-1. (**J**) Results of quantitative RT-PCR and (**K**) Western blot assay showing the mucosal expression of p47phox mRNA and protein, respectively. Scale bar, 100 μm. **P < 0.01, ***P < 0.001 vs. WT. #*P* < 0.05, ###*P* < 0.001 vs. WT + TNBS

We then detected the effects of Atox1 on inflammation in TNBS-induced colitis using mice homozygous for the conditional Atox1 allele (Atox1^−/−^); WT mice were used as control. TNBS treatment led to a decrease in body weight and an increase in DAI scores in WT mice (Fig. 4A-B). However, these TNBS-induced changes were largely ameliorated in Atox1^−/−^ mice models (Fig. 4A-B). Histological analysis showed that TNBS-treated Atox1^−/−^ mice had ameliorated intestinal inflammation, increased villus height, and preserved intestinal crypt architecture compared to TNBS-treated WT mice (Fig. 4C). Following TNBS treatment, IL-6, TNF-α, IFN-γ, and IL-17 levels were significantly elevated in the colon tissue of WT mice, but were lower in Atox1^−/−^ mice models (Fig. 4D-G). TNBS treatment led to an increase in mRNA levels of iNOS and IL-12p40 but a decrease in mRNA levels of IL-10 and Arg-1 in the intestinal mucosa of WT mice, and these effects were attenuated in Atox1^−/−^ mice models (Fig. 4H-I). TNBS-treated WT mice showed upregulation of both mRNA and protein levels of p47phox (Fig. 4J-K). Atox1 knockout in mice suppressed TNBS-induced p47phox up-regulation (Fig. 4J-K). These results demonstrate that Atox1 knockout attenuated the development of colitis in mice by alleviating inflammation and suppressing M1 polarization of macrophages.

### Atox1 knockout inhibited pro-inflammatory cytokines and macrophage M1 polarization

**Fig. 5:**
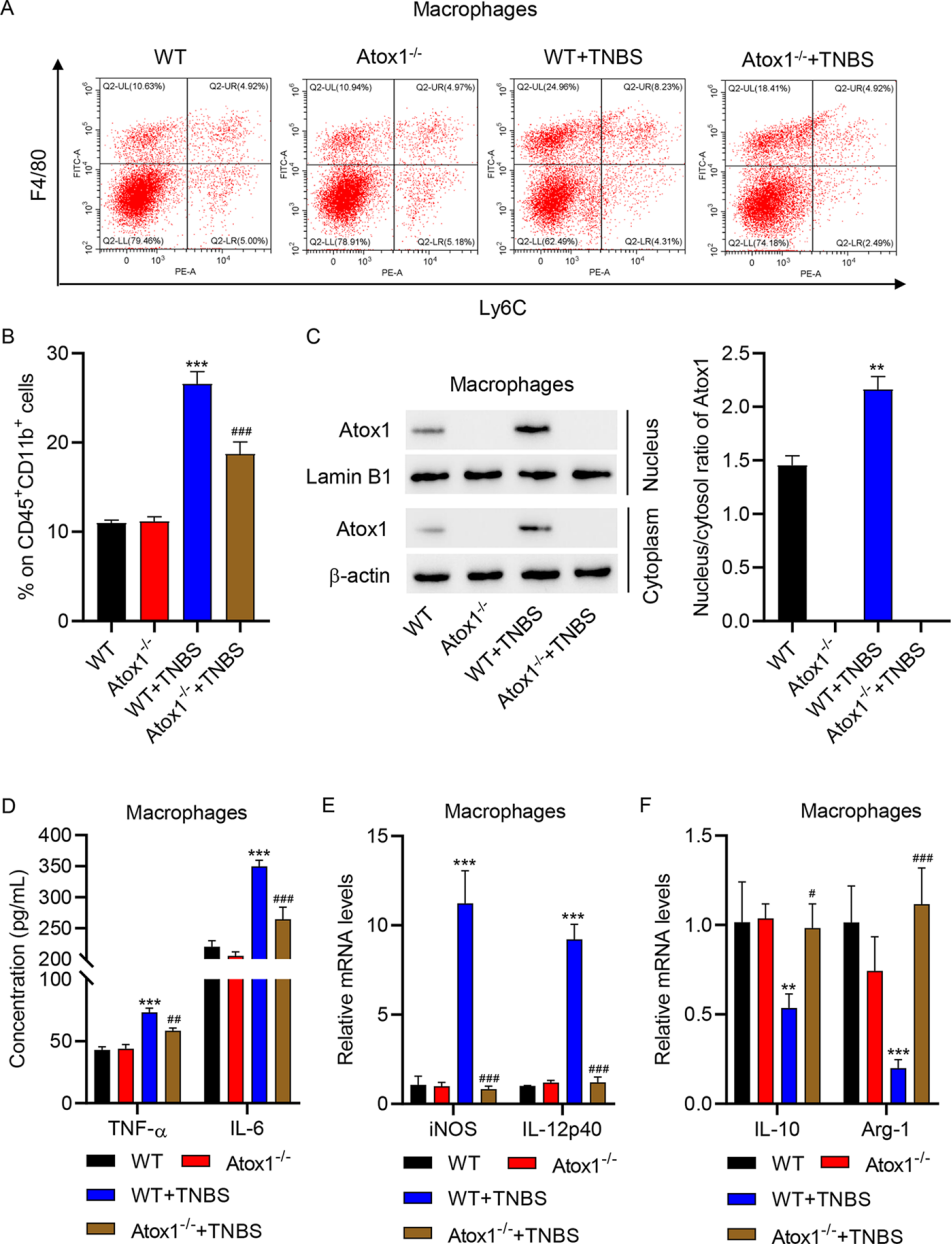
Atox1 knockout inhibited macrophage M1 polarization. (**A**, **B**) Flow cytometry analysis of the percentage of macrophages (CD45^+^CD11b^+^F4/80^+^Ly6C^−^) among the colonic lamina propria mononuclear cells isolated from Atox1^−/−^ and WT mice with or without TNBS. Atox1^−/−^ mice and WT littermates received TNBS and the macrophages were isolated. (**C**) Western blot assay of Atox1 expression. (**D**) Results of ELISA showing the levels of pro-inflammatory cytokines TNF-α and IL-6. Results of quantitative RT-PCR showing the mRNA expressions of (**E**) iNOS, (**E**) IL-12p40, (**F**) IL-10 and (**F**) Arg-1. **P < 0.01, ***P < 0.001 vs. WT. #*P* < 0.05, ##*P* < 0.01, ###*P* < 0.001 vs. WT + TNBS

To investigate the impact of Atox1 on macrophages in colitis, the percentage of macrophages among colonic lamina propria mononuclear cells isolated from Atox1^−/−^ and WT mice with or without TNBS induction was measured by flow cytometry. As shown in Fig. 5A-B, after TNBS induction, Atox1^−/−^ mice showed a decreased percentage of macrophages compared with WT mice. Atox knockout in macrophages was observed in Atox1^−/−^ mice with or without TNBS induction (Fig. 5C). TNBS promoted the production of TNF-α and IL-6, which was partly suppressed by Atox1 knockout (Fig. 5D). TNBS induction increased the mRNA levels of iNOS and IL-12p40 but decreased the mRNA levels of IL-10 and Arg-1, an effect which was largely reversed by Atox1 knockout (Fig. 5E,F).

### Atox1 participated in regulating macrophage polarization by affecting ROS production and NLRP3 inflammasome

**Fig. 6:**
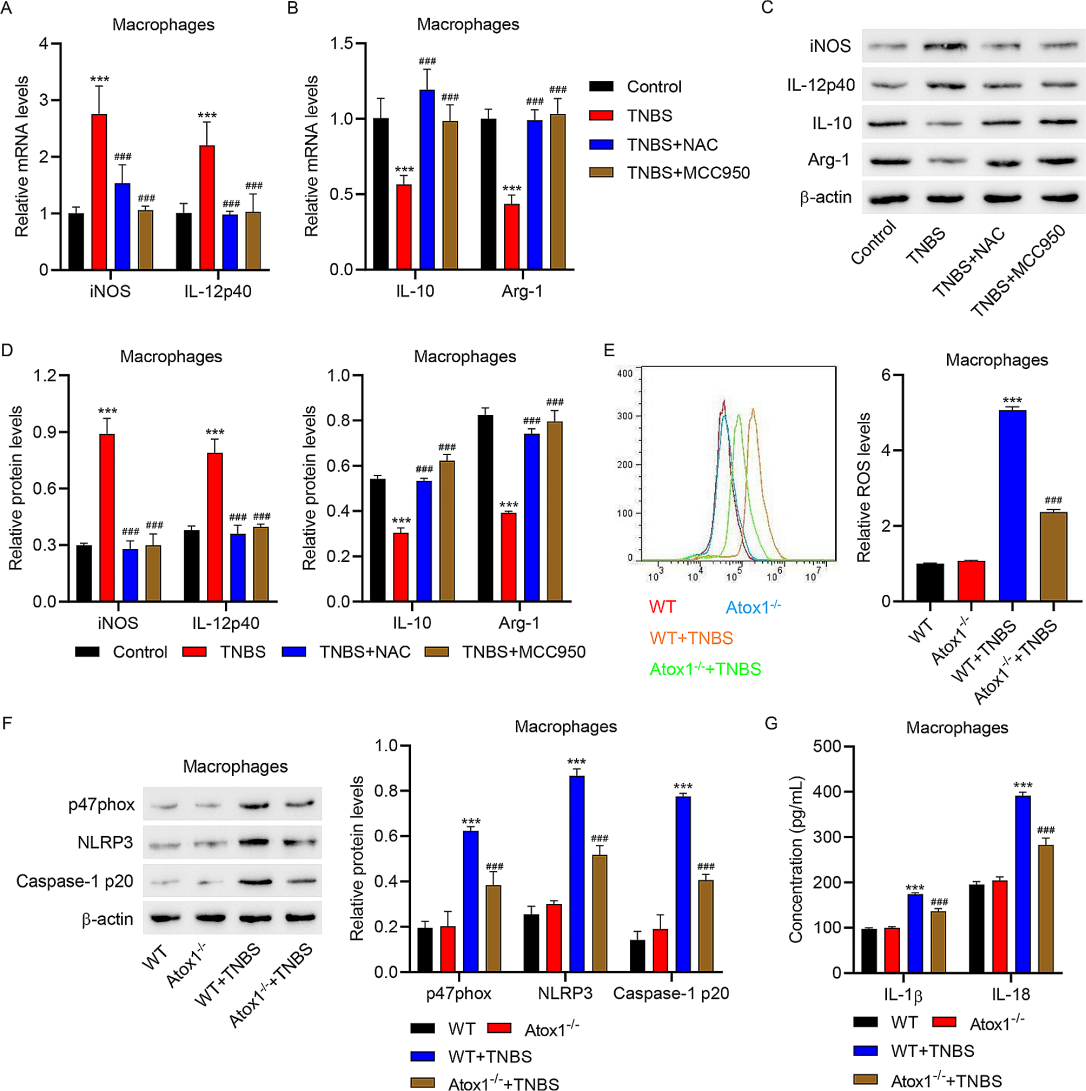
Atox1 knockout inhibited ROS production and NLRP3 inflammasome activation in macrophages. Macrophages isolated from the intestinal mucosa of control and TNBS-induced mice were treated with NAC or MCC950. (**A**, **B**) Quantitative RT-PCR and (**C**, **D**) Western blot analyses of the expression of iNOS, IL-12p40, IL-10, and Arg-1. Macrophages were isolated from Atox1^−/−^ mice and WT littermates with or without TNBS treatment. (**E**) Flow cytometry analysis of ROS production. (**F**) Western blot assay to estimate the protein expressions of p47phox, NLRP3, and Caspase-1 p20. (**G**) Results of ELISA showing the levels of pro-inflammatory cytokines IL-1β and IL-18. ***P < 0.001 vs. control or WT. ###*P* < 0.001 vs. TNBS or WT + TNBS

Macrophages isolated from the intestinal mucosa of control or TNBS-induced mice were treated with NAC or MCC950 to investigate the effects of Atox1 on ROS production and NLRP3 inflammasome activation. Macrophages isolated from TNBS-induced mice showed higher mRNA and protein levels of iNOS and IL-12p40, and lower mRNA and protein levels of IL-10 and Arg-1 compared to those isolated from control mice; these effects were restored by NAC or MCC950 treatment (Fig. 6A-D). These findings indicated the involvement of ROS production and NLRP3 inflammasome activation in regulating macrophage polarization. TNBS induction led to ROS accumulation in macrophages, an effect that was abrogated by Atox1 knockout (Fig. 6E). TNBS treatment resulted in up-regulation of p47phox, NLRP3, and Caspase-1 p20 proteins in macrophages, an effect that was counteracted by Atox1 knockout (Fig. 6F). Moreover, TNBS-induced increased concentrations of pro-inflammatory IL-1β and IL-18 were partly restored by Atox1 knockout in macrophages (Fig. 6G). Collectively, these observations imply a potential role of Atox1 in regulating ROS production and NLRP3 inflammasome activation, thus affecting the polarization of macrophages.

### Atox1 knockout suppressed M1 polarization of macrophages, pro-inflammatory cytokines, and NLRP3 inflammasome activation in TNBS-induced colitis mice

**Fig. 7:**
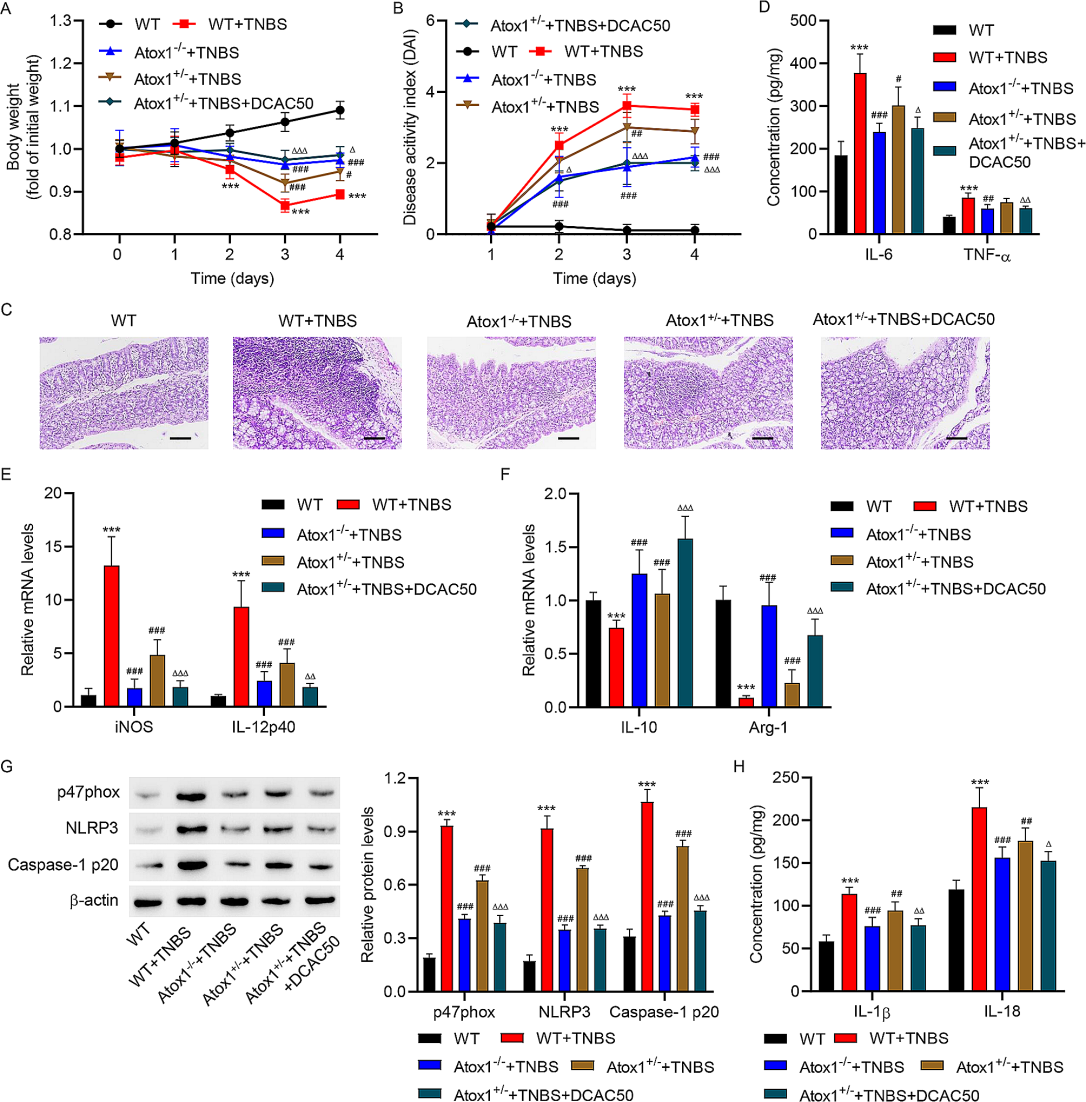
Atox1 knockout decreased pro-inflammatory cytokines and NLRP3 inflammasome activation in TNBS-induced colitis mice. Atox1^−/−^ and Atox1^+/−^ mice and WT littermates received TNBS with or without DCAC50 treatment. Colitis induction was evaluated by (**A**) body weight loss, expressed as a percentage of the initial weight, and (**B**) clinical DAI. (**C**) Representative images of histological analysis. (**D**) Results of ELISA showing the mucosal production of the pro-inflammatory cytokines TNF-α and IL-6. Results of quantitative RT-PCR showing the mucosal mRNA expression of (**E**) iNOS, (**E**) IL-12p40, (**F**) IL-10 and (**F**) Arg-1. (**G**) Western blot assay to estimate the mucosal expression of p47phox, NLRP3, and Caspase-1 p20 protein; (**H**) Results of ELISA showing the levels of pro-inflammatory cytokines IL-1β and IL-18. Scale bar, 100 μm. ***P < 0.001 vs. WT. #*P* < 0.05, ##*P* < 0.01, ###*P* < 0.001 vs. WT + TNBS. Δ*P* < 0.05, ΔΔ*P* < 0.01, ΔΔΔ*P* < 0.001 vs. Atox1^+/−^+TNBS

The Atox1 expressions in colonic tissues of WT, Atox1 heterozygous knockout (Atox1^+/−^), and Atox1 homozygous knockout (Atox1^−/−^) mice with or without TNBS treatment are shown in Supplementary Fig. 2. TNBS treatment induced a decrease in body weight and an increase in clinical DAI scores in WT mice, an effect that was alleviated by Atox1 knockout (Fig. 7A-B). Histological analysis showed that following TNBS treatment, Atox1^+/−^ and Atox1^−/−^ mice had ameliorated intestinal inflammation, increased villus height, and preserved intestinal crypt architecture compared to TNBS-treated WT mice (Fig. 7C). Following TNBS treatment, Atox1^+/−^ and Atox1^−/−^ mice also showed decreased mucosal production of pro-inflammatory TNF-α and IL-6 and mRNA levels of iNOS and IL-12p40, and increased mRNA levels of IL-10 and Arg-1 compared to TNBS-induced WT mice (Fig. 7D-F). Moreover, TNBS-induced up-regulation of p47phox, NLRP3, Caspase-1 p20, IL-1β, and IL-18 in WT mice were attenuated in Atox1^+/−^ and Atox1^−/−^ mice (Fig. 7G-H). Furthermore, the effect of Atox1^+/−^ was weaker than Atox1^−/−^ and inhibited by DCAC50 (Fig. 7A-H). TNBS-induced intestinal inflammation and expressions of p47phox, NLRP3, and Caspase-1 p20 in colonic tissues were also inhibited by DCAC50 (Supplementary Fig. 3A-B).

## Discussion

**Fig. 8:**
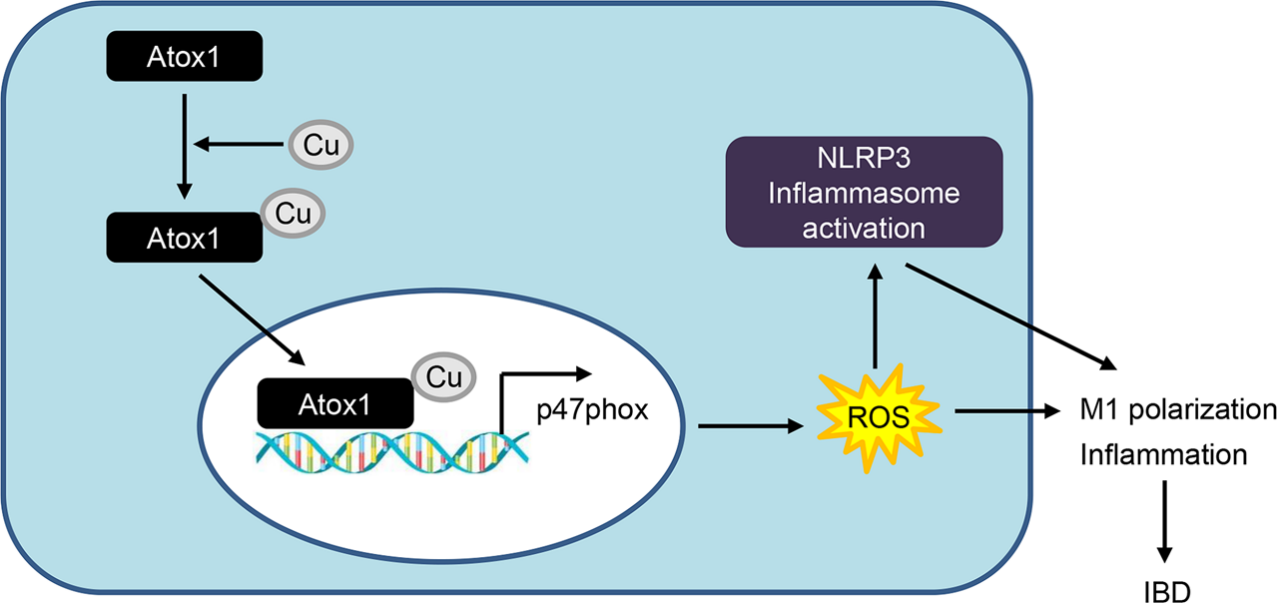
Schematic illustration of Atox1 function in regulating ROS-NLRP3 inflammasome pathway during IBD

IBD is characterized by immune dyshomeostasis in the digestive tract [31]. Macrophages play a critical role in intestinal immune homeostasis and have been identified as a potential therapeutic target for IBD [32]. Our study highlighted the relationship between Atox1 and macrophages in the pathogenesis of IBD. Atox1 was found to promote M1 polarization of macrophages and increase the concentrations of pro-inflammatory cytokines in intestinal tissue by regulating the ROS-NLRP3 inflammasome pathway (Fig. 8), suggesting its important role in the pathogenesis of IBD. TNBS elicits a colitis-like phenotype with histopathological and morphological changes mimicking Crohn’s disease [22, 23]. The results regarding body weight, DAI, and histological staining on day 3 after TNBS induction were consistent with those on day 4, suggesting that the peak intestinal inflammation activity was reached on day 3 and that mice might have begun adapting to the insult by day 4. These findings were consistent with those of previous studies [22, 24].

A previous study has implicated the *Atox1* gene in the causation of ulcerative colitis [15]. Similarly, our study demonstrated the upregulation of Atox1 in patients with Crohn’s disease and the TNBS-induced mouse model of Crohn’s disease. Our observations are consistent with previous studies which showed that Atox1 promotes macrophage M1 polarization and production of pro-inflammatory cytokines. These findings indicate that macrophages represent a novel therapeutic target in IBD given their role in promoting intestinal inflammation [33, 34]. Similarly, Cu has been shown to promote M1 polarization of macrophages [20]. However, a lower concentration of Cu was found to promote the expression of M2 polarization-related genes, indicating a concentration-dependent effect of Cu [21]. Cu also induces inflammation through the NF-κB pathway [9]. In addition, NF-κB, a crucial transcription factor regulating inflammatory responses [35], may induce the upregulation of copper transporter CTR1, which in turn promotes Cu uptake and affects the Atox1 translocation or transcriptional activity, suggesting a potential mutual regulation between inflammation and Atox1.

Recent studies have suggested a pivotal role of ROS in the pathophysiology of IBD and its potential as a therapeutic target to curb disease progression [2, 6]. Our observations are consistent with previous studies showing markedly decreased ROS levels in Atox1^−/−^ mice [16, 36, 37]. However, in addition to its role as a copper chaperone, Atox1 is known to protect against oxidative stress. For example, Tat fusion protein Tat-ATOX1 was shown to inhibit ROS production in STZ-exposed RINm5F cells [38] and LPS-induced Raw 264.7 cells [39]. Our observations are consistent with previous studies in which Atox1 knockout reduced p47phox, ROS production, and M1 polarization, suggesting the important implications of the Atox1-p47phox-ROS-M1 polarization axis in modulating inflammation during the progression of IBD [16, 40, 41]. NLRP3 inflammasomes are implicated in the secretion of IL-1β and IL-18 and Caspase-1-mediated pyroptosis, a potential programmed cell death process [42], and associated with the onset and progression of IBD [43]. In the present study, Atox1 knockout in TNBS-induced mice inhibited ROS-induced NLRP3 inflammasome activation. These findings are consistent with previous studies in which Cu was shown to promote pyroptosis by inducing ROS production, leading to the formation of the NLRP3 inflammasome, and suggesting the involvement of the Atox1-induced trafficking in ROS-induced NLRP3 inflammasome activation [43, 44]. Inhibition of Atox1 activity with DCAC50 [25] inhibited CuCl_2_-induced p47phox expression and macrophage M1 polarization and the therapeutic effects of Atox1 knockout in TNBS-induced mice, suggesting the involvement of copper chaperone-induced Cu trafficking in TNBS-induced colitis.

In addition to functioning as a Cu-dependent transcription factor for p47phox, Cu chaperone Atox1 was also associated with intracellular Cu-trafficking by transferring Cu to ATP7A that delivers Cu to Cu-containing secretory enzymes and functions in the Cu egress to maintain the level of intracellular Cu [45]. Our results showed that the Atox1 protein expression was increased while Cu content was decreased in colonic tissues and macrophages of TNBS-induced colitis mice. These data suggested that TNBS-induced Atox1 upregualtion may promote Cu efflux bytransferring Cu to ATP7A. Moreover, Cu supplement could polarize macrophages to pro-inflammatory M1 phenotype by activating Cu-transport signaling such as ATP7A in macrophages [46], suggesting that ATP7A-mediated Cu efflux may also involve in the macrophage polarization, contributing to the inflammation of IBD.

Some limitations of this study should be acknowledged. First, we did not verify Atox1 expression levels in clinical specimens from patients with Crohn’s disease and only performed bioinformatics analysis of clinical data. Second, there is a need to perform RNA-sequencing and pathway-enrichment analysis to elucidate the role of Atox1 in macrophages during the inflammatory process. Third, further experiments are required to validate the roles of the ROS-NLRP3 inflammasome pathway in mediating the impact of Atox1 on macrophage polarization. In addition, DCAC50 inhibits not only Atox1 but also Cu chaperone CCS. Thus, the potential involvement of other Cu chaperones in inducing macrophage polarization and inflammation in IBD cannot be ruled out. Future studies should also investigate the in vivo role of nuclear Atox1 in other IBD animal models such as dextran sodium sulfate-induced ulcerative colitis. Although Cu trafficking contributes to inflammation and the onset of IBD, translating the findings from animal models to humans has inherent limitations. Therefore, it is imperative to establish an alternative Cu trafficking model to gain further insights into the molecular mechanisms of IBD and guide therapeutic strategies.

## Conclusions

We report Atox1 up-regulation in colon tissues of TNBS-induced colitis mice models. Using in vitro and in vivo experiments, our study suggests that pro-inflammatory Atox1 promotes M1 polarization of macrophage and production of pro-inflammatory cytokines in intestines through the ROS-NLRP3 inflammasome pathway. This study advances our understanding of the biological effects of Atox1 and the underlying mechanisms in IBD. Targeting Atox1 may represent a novel ROS-dependent therapeutic approach for IBD.

### Electronic supplementary material

Below is the link to the electronic supplementary material.

Supplementary Material 1

## Data Availability

The datasets used and/or analysed during this study were accessed via the corresponding authors on reasonable request.

## Abbreviations

IBD: Inflammatory bowel disease
Atox1: Antioxidant-1
TNBS: 2,4,6-Trinitrobenzenesulfonic acid
WT: Wild type
DAI: Disease activity index
H&E: Hematoxylin and eosin
NAC: N-acetyl cysteine
UMAP: Uniform Manifold Approximation and Projection

## References

[1] M’Koma AE (2022). Inflammatory bowel disease: clinical diagnosis and surgical treatment-overview. Med (Kaunas).

[2] Bourgonje AR, Feelisch M, Faber KN, Pasch A, Dijkstra G, van Goor H (2020). Oxidative stress and redox-modulating therapeutics in inflammatory bowel disease. Trends Mol Med.

[3] Tarris G, de Rougemont A, Charkaoui M, Michiels C, Martin L, Belliot G (2021). Enteric viruses and inflammatory bowel disease. Viruses.

[4] Agrawal M, Allin KH, Colombel JF, Jess T, Petralia F (2022). Multiomics to elucidate inflammatory bowel disease risk factors and pathways. Nat Rev Gastroenterol Hepatol.

[5] Li J, Qiu H, Gong H, Tong W (2021). Broad-spectrum reactive oxygen species scavenging and activated macrophage-targeting microparticles ameliorate inflammatory bowel disease. Biomacromolecules.

[6] Zhang J, Cen L, Zhang X, Tang C, Chen Y, Zhang Y (2022). Mpst deficiency promotes intestinal epithelial cell apoptosis and aggravates inflammatory bowel disease via akt. Redox Biol.

[7] Jarmakiewicz-Czaja S, Ferenc K, Filip R (2023). Antioxidants as protection against reactive oxidative stress in inflammatory bowel disease. Metabolites.

[8] Liao Y, Zhao J, Bulek K, Tang F, Chen X, Cai G (2020). Inflammation mobilizes copper metabolism to promote colon tumorigenesis via an Il-17-steap4-xiap axis. Nat Commun.

[9] Zhou Q, Zhang Y, Lu L, Zhang H, Zhao C, Pu Y (2022). Copper induces microglia-mediated neuroinflammation through ros/nf-κb pathway and mitophagy disorder. Food Chem Toxicol.

[10] Maung MT, Carlson A, Schachtschneider KM, Padilla-Benavides T, Olea-Flores M, Navarro-Tito N (2021). The molecular and cellular basis of copper dysregulation and its relationship with human pathologies. FASEB J.

[11] Yang D, Xiao P, Qiu B, Yu HF, Teng CB (2023). Copper chaperone antioxidant 1: multiple roles and a potential therapeutic target. J Mol Med.

[12] Tsymbal S, Refeld A, Zatsepin V, Kuchur O (2023). The p53 protein is a suppressor of atox1 copper chaperon in tumor cells under genotoxic effects. PLoS ONE.

[13] Deng R, Zhu L, Jiang J, Chen J, Li H. Cuproptosis-related gene lipt1 as a prognostic indicator in non-small cell lung cancer: functional involvement and regulation of atox1 expression. Biomol Biomed. 2023. 10.17305/bb.2023.9931 PMC11088889 38041690

[14] Jana A, Das A, Krett NL, Guzman G, Thomas A, Mancinelli G (2020). Nuclear translocation of atox1 potentiates activin a-induced cell migration and colony formation in colon cancer. PLoS ONE.

[15] Zou M, Zhang W, Zhu Y, Xu Y (2023). Identification of 6 cuproptosis-related genes for active ulcerative colitis with both diagnostic and therapeutic values. Medicine.

[16] Das A, Sudhahar V, Ushio-Fukai M, Fukai T (2019). Novel interaction of antioxidant-1 with traf4: role in inflammatory responses in endothelial cells. Am J Physiol Cell Physiol.

[17] Zhou X, Li W, Wang S, Zhang P, Wang Q, Xiao J (2019). Yap aggravates inflammatory bowel disease by regulating m1/m2 macrophage polarization and gut microbial homeostasis. Cell Rep.

[18] Jiang H, Dong J, Li Y, Yang X, Lu Q, Li J (2022). Qingchang wenzhong decoction alleviates dss-induced inflammatory bowel disease by inhibiting m1 macrophage polarization in vitro and in vivo. BioMed Res Int.

[19] Seyedizade SS, Afshari K, Bayat S, Rahmani F, Momtaz S, Rezaei N (2020). Current status of m1 and m2 macrophages pathway as drug targets for inflammatory bowel disease. Arch Immunol Ther Exp.

[20] Xu D, Zhu W, Ding C, Mei J, Zhou J, Cheng T (2023). Self-homeostasis immunoregulatory strategy for implant-related infections through remodeling redox balance. ACS Nano.

[21] Díez-Tercero L, Delgado LM, Bosch-Rué E, Perez RA (2021). Evaluation of the immunomodulatory effects of cobalt, copper and magnesium ions in a pro inflammatory environment. Sci Rep.

[22] Hasanpour H, Falak R, Mokhtarian K, Sadeghi F, Masoumi E, Asadollahi P (2024). The effects of fasciola hepatica recombinant proteins (peroxiredoxin and cathepsin l1) on crohn’s disease experimental model. Parasite Immunol.

[23] Ortiz-Cerda T, Argüelles-Arias F, Macías-García L, Vázquez-Román V, Tapia G, Xie K (2023). Effects of polyphenolic maqui (aristotelia chilensis) extract on the inhibition of nlrp3 inflammasome and activation of mast cells in a mouse model of Crohn’s disease-like colitis. Front Immunol.

[24] Török S, Almási N, Veszelka M, Börzsei D, Szabó R, Varga C. Protective effects of h(2)s donor treatment in experimental colitis: a focus on antioxidants. Antioxidants. 2023;12. 10.3390/antiox12051025 PMC10215296 37237891

[25] Karginova O, Weekley CM, Raoul A, Alsayed A, Wu T, Lee SS (2019). Inhibition of copper transport induces apoptosis in triple-negative breast cancer cells and suppresses tumor angiogenesis. Mol Cancer Ther.

[26] Bi Z, Cui E, Yao Y, Chang X, Wang X, Zhang Y (2022). Recombinant bifidobacterium longum carrying endostatin protein alleviates dextran sodium sulfate-induced colitis and colon cancer in rats. Front Microbiol.

[27] Genua M, D’Alessio S, Cibella J, Gandelli A, Sala E, Correale C (2015). The urokinase plasminogen activator receptor (upar) controls macrophage phagocytosis in intestinal inflammation. Gut.

[28] Tang J, Liu J, Yan Q, Gu Z, August A, Huang W (2021). Konjac Glucomannan oligosaccharides prevent intestinal inflammation through signr1-mediated regulation of alternatively activated macrophages. Mol Nutr Food Res.

[29] Itoh S, Kim HW, Nakagawa O, Ozumi K, Lessner SM, Aoki H (2008). Novel role of antioxidant-1 (atox1) as a copper-dependent transcription factor involved in cell proliferation. J Biol Chem.

[30] Martin JC, Chang C, Boschetti G, Ungaro R, Giri M, Grout JA (2019). Single-cell analysis of Crohn’s disease lesions identifies a pathogenic cellular module associated with resistance to anti-tnf therapy. Cell.

[31] Bisgaard TH, Allin KH, Keefer L, Ananthakrishnan AN, Jess T (2022). Depression and anxiety in inflammatory bowel disease: Epidemiology, mechanisms and treatment. Nat Rev Gastroenterol Hepatol.

[32] Pan X, Zhu Q, Pan LL, Sun J (2022). Macrophage immunometabolism in inflammatory bowel diseases: from pathogenesis to therapy. Pharmacol Ther.

[33] Jiang H, Wu X, Zhao Y, Li Y, Liu J, Gong W (2022). Macrophages-microenvironment crosstalk in fibrostenotic inflammatory bowel disease: from basic mechanisms to clinical applications. Expert Opin Ther Targets.

[34] Bain CC, Hegarty LM, Jones GR (2023). Macrophages in intestinal homeostasis and inflammatory bowel disease. Nat Rev Gastroenterol Hepatol.

[35] Zeng L, Wang YH, Ai CX, Zhang JS (2018). Differential effects of β-glucan on oxidative stress, inflammation and copper transport in two intestinal regions of large yellow croaker larimichthys crocea under acute copper stress. Ecotoxicol Environ Saf.

[36] Das A, Sudhahar V, Chen GF, Kim HW, Youn SW, Finney L (2016). Endothelial antioxidant-1: a key mediator of copper-dependent wound healing in vivo. Sci Rep.

[37] Chen GF, Sudhahar V, Youn SW, Das A, Cho J, Kamiya T (2015). Copper transport protein antioxidant-1 promotes inflammatory neovascularization via chaperone and transcription factor function. Sci Rep.

[38] Ahn EH, Kim DW, Shin MJ, Ryu EJ, Yong JI, Chung SY (2016). Tat-atox1 inhibits streptozotocin-induced cell death in pancreatic rinm5f cells and attenuates diabetes in a mouse model. Int J Mol Med.

[39] Kim DW, Shin MJ, Choi YJ, Kwon HJ, Lee SH, Lee S (2018). Tat-atox1 inhibits inflammatory responses via regulation of mapk and nf-κb pathways. BMB Rep.

[40] Han C, Sheng Y, Wang J, Zhou X, Li W, Zhang C (2022). Nox4 promotes mucosal barrier injury in inflammatory bowel disease by mediating macrophages m1 polarization through ros. Int Immunopharmacol.

[41] Shi L, Zhang P, Jin R, Chen X, Dong L, Chen W (2022). Dioscin ameliorates inflammatory bowel disease by up-regulating mir-125a-5p to regulate macrophage polarization. J Clin Lab Anal.

[42] Zhang Y, Yang W, Li W, Zhao Y (2021). Nlrp3 inflammasome: checkpoint connecting innate and adaptive immunity in autoimmune diseases. Front Immunol.

[43] Chen QL, Yin HR, He QY, Wang Y (2021). Targeting the nlrp3 inflammasome as new therapeutic avenue for inflammatory bowel disease. Biomed Pharmacother.

[44] Xue Q, Kang R, Klionsky DJ, Tang D, Liu J, Chen X (2023). Copper metabolism in cell death and autophagy. Autophagy.

[45] Kamiya T (2022). Copper in the tumor microenvironment and tumor metastasis. J Clin Biochem Nutr.

[46] Huang Q, Ouyang Z, Tan Y, Wu H, Liu Y (2019). Activating macrophages for enhanced osteogenic and bactericidal performance by Cu ion release from micro/nano-topographical coating on a titanium substrate. Acta Biomater.

