# Supplementary material for: Atox1 regulates macrophage polarization in intestinal inflammation via ROS-NLRP3 inflammasome pathway
Source: J Transl Med. 2024 May 25;22:497. doi: 10.1186/s12967-024-05314-4 (PMC11128112; doi:10.1186/s12967-024-05314-4)
Supplement: Supplementary file 1 — Supplementary Material 1 [file 12967_2024_5314_MOESM1_ESM.docx]

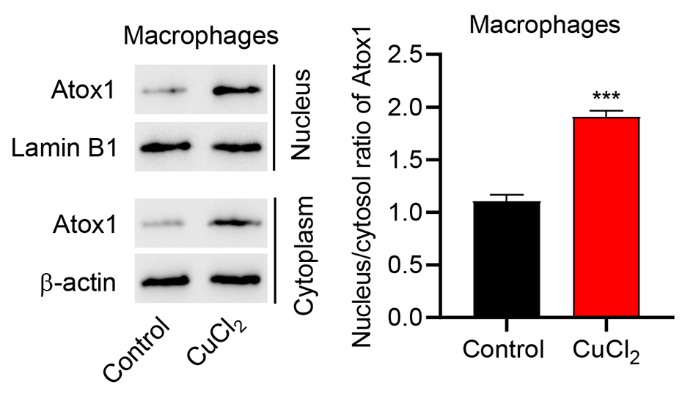


Supplementary Figure 1. Western blot analysis of the Atox1 expression in macrophages isolated from the intestinal mucosa of mice and treated with or without CuCl_2_. ****P*<0.001 *vs* control.


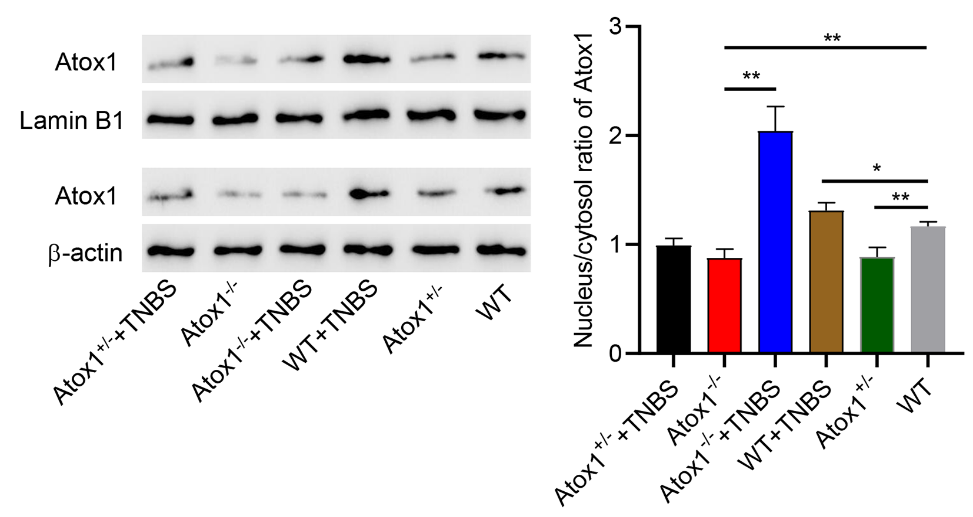


Supplementary Figure 2. Atox1^-/-^ and Atox1^+/-^ mice and WT littermates received TNBS with or without DCAC50 treatment, and the mucosal expression of Atox1 expression was detected by Western blot analysis. **P*<0.05, ***P*<0.01.


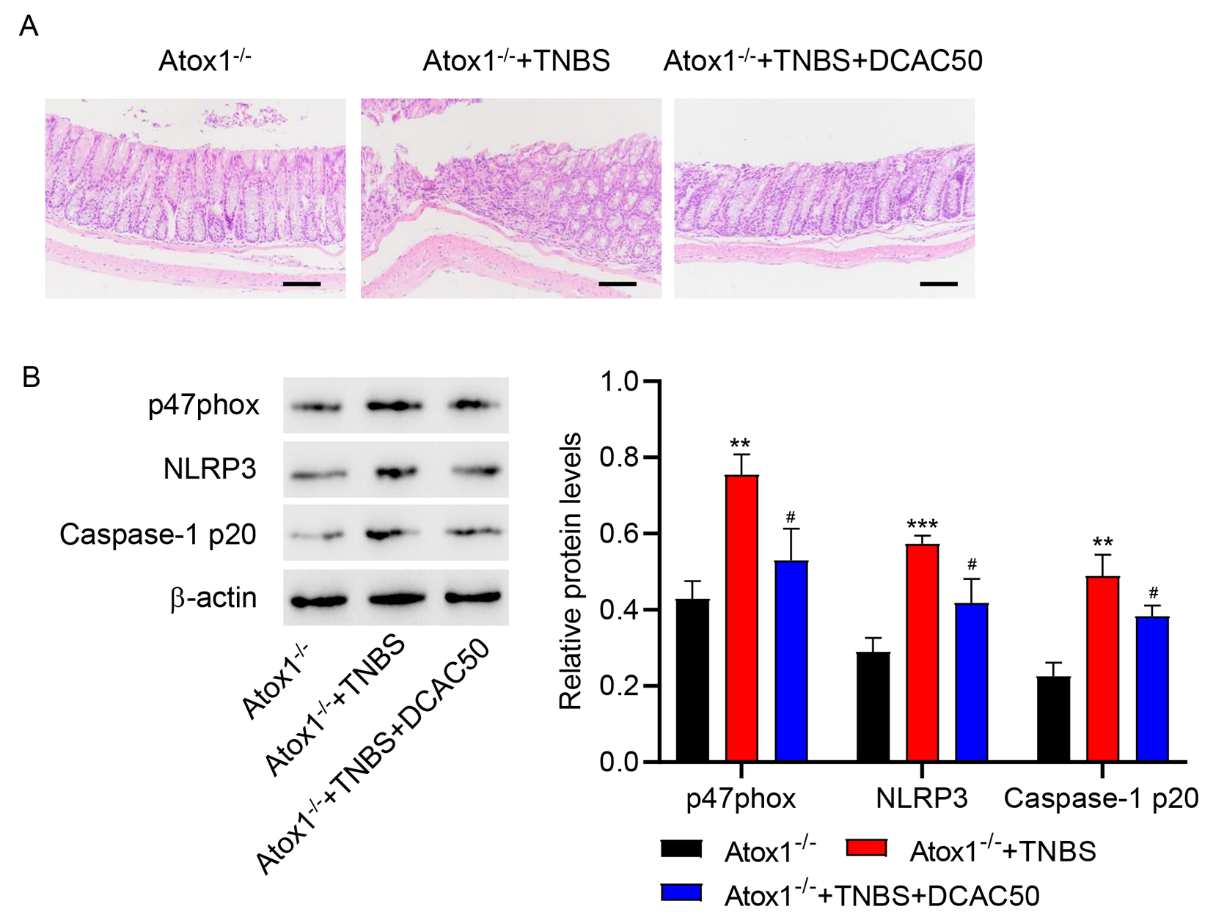


Supplementary Figure 3. Atox1^-/-^ mice received TNBS with or without DCAC50 treatment. (A) Representative images of histological analysis. (B) Western blot analysis of the mucosal expression of p47phox, NLRP3, and Caspase-1 p20 protein. Scale bar, 100 μm. ****P* < 0.001 *vs* Atox1^-/-^. #*P*<0.05 *vs* Atox1^-/-^+TNBS.
